# Comparison of the effectiveness of single-dose levofloxacin with single-dose fosfomycin pre-urodynamic study related to the incidence of urinary tract infection: a randomized controlled trial

**DOI:** 10.1186/s12894-025-01839-y

**Published:** 2025-07-14

**Authors:** Harrina Erlianti Rahardjo, Fina Widia, Cindy Wijaya, Kevin Leonardo, Alfred Tanjung, Muhammad Hanif Arfiananda, Fatimah Nuwwaaridya Fitriani, Rahmat Aidil Fajar Siregar, Andika Afriansyah

**Affiliations:** 1 Department of Urology, Faculty of Medicine, Universitas Indonesia – Cipto Mangunkusumo Hospital, Jakarta, Indonesia; 2 Division of Urology, Department of Surgery, Persahabatan Hospital - Faculty of Medicine, Universitas Indonesia, Jakarta, Indonesia

**Keywords:** Antibiotic, Levofloxacin, Fosfomycin, Prophylaxis, Randomized trial, Urinary tract infection, Urodynamic study

## Abstract

**Purpose:**

The purpose of this study is to examine the efficacy of 500 mg levofloxacin and 3 g fosfomycin given as single-dose prophylactic antibiotics one hour before urodynamic studies (UDS) to prevent urinary tract infections (UTIs).

**Methods:**

This single-blinded, randomized clinical trial included 126 patients who underwent UDS between December 2022 and March 2024 at two urology centers in Jakarta. The patients, aged 18 years and older, were randomized equally into two groups receiving either 500 mg levofloxacin or 3 g fosfomycin prior to UDS. The primary indications for UDS included lower urinary tract symptoms (LUTS) (54.8%), overactive bladder (OAB) (32.8%), underactive bladder (8.9%), and stress urinary incontinence (3.5%). Three days post-UDS, patients were followed up with urinalysis and clinical assessment for UTI symptoms.

**Results:**

Among the 126 patients, UTI was diagnosed in 26 cases (20.9%), with 14 cases (22.2%) in the levofloxacin group and 12 cases (19%) in the fosfomycin group, showing no significant difference (*p* = 0.660). Symptomatic UTIs occurred in 6 patients (4.8%) in the levofloxacin group and 7 patients (5.6%) in the fosfomycin group. E. coli was the most commonly detected bacterium in urine cultures.

**Conclusion:**

In our center, there was no significant difference in the clinical outcome between the administration of 3 g fosfomycin and 500 mg levofloxacin as single-dose prophylactic antibiotics prior to UDS. Given the rising antibiotic resistance, fosfomycin may be considered an alternative. Further multicenter and multinational studies are warranted, as bacterial profiles and antibiotic resistance patterns may vary across different regions and healthcare systems.

**Trial registration:**

NCT06017479.

**Study Registration Date:**

2023-08-24.

**Supplementary Information:**

The online version contains supplementary material available at 10.1186/s12894-025-01839-y.

## Background

Urodynamic study (UDS) is an important examination in urology, often used to assist in diagnosing lower urinary tract functional disorders. The frequency of this examination has consistently increased every year [1]. However, due to its invasive nature, UDS poses a risk of post-examination urinary tract infections (UTIs) [2]. The incidence rate of post-UDS UTI varies between 1.5–30% 3. Given this risk factor, patients undergoing UDS, especially those with a high risk of UTIs, are recommended to receive prophylactic antibiotics [4]. In their research, Rahardjo et al. found that the use of levofloxacin as post-UDS prophylactic antibiotic for 3 days can reduce the risk of UTI by 55% compared to placebo. However, the study observed no difference between using a single dose of 500 mg levofloxacin pre-UDS and 500 mg levofloxacin for 3 days post-UDS [5]. 

The most common etiologic pathogen of UTI is *E. coli* [6, 7]. Data from the Clinical Pathology Department of Dr. Cipto Mangunkusumo National Referral Hospital from July to December 2021 revealed that 33% of 415 *E. coli* isolates cultured from urine specimens were sensitive to levofloxacin, a figure that is lower when compared to fosfomycin, where a sensitivity rate of 84% was found in 219 *E. coli* isolates [8]. Oral levofloxacin has been the primary antibiotic used before urodynamic studies to prevent post-UDS UTI [5, 9]. However, the increased use of fluoroquinolone antibiotics has led to a decrease in levofloxacin sensitivity [10]. Levofloxacin resistance among bacterial isolates in Indonesian hospitals is notable, with 35.8% of isolates showing resistance [11]. Levofloxacin resistance rates began at 1.2% in 1988 and have progressively increased to 25% by the end of 2014, reaching 38.1% by 2017 in the United States [10]. 

Levofloxacin resistance is a growing concern, particularly for patients undergoing urodynamic studies. Understanding local resistance patterns and employing appropriate strategies for prevention and treatment can help manage the risks associated with infections in this population. This study aimed to compare the effectiveness of using a single dose of 500 mg levofloxacin and a single dose of 3 g fosfomycin, administered one hour prior to a UDS examination as prophylactic antibiotics to prevent UTIs.

## Methods

### Study population

In order to compare the number of UTIs in a group of patients who received a single dosage of 500 mg of levofloxacin and patients who received 3 g of fosfomycin one hour before UDS, this experimental investigation used a single-blinded randomized clinical trial design. Between December 2022 and March 2024, the study was conducted in two outpatient urology centers located in Jakarta: Dr. Cipto Mangunkusumo General Hospital and Persahabatan General Hospital.

Inclusion criteria for the study consist of male or female patients over 18 years of age who are indicated for urodynamic testing and have expressed a willingness to participate in the study. Exclusion criteria include an allergy to levofloxacin or fosfomycin, pregnancy, uncontrolled diabetes mellitus, use of an indwelling urinary catheter, presence of a UTI prior to urodynamic testing as determined by clinical symptoms and urine examination results, and refusal to participate in the study.

The sample size was calculated based on a comparison of two independent proportions using the formula described in Fundamentals of Clinical Research Methodology. We assumed a 20% absolute difference in the incidence of post-UDS bacteriuria between groups receiving levofloxacin (P₁ = 0.7) and fosfomycin (P₂ = 0.9), with α = 0.05 and 80% power (Zα = 1.96; Zβ = 0.842). Based on this, the minimum sample size required was 62 patients per group. Anticipating a small dropout rate, the final total sample size was set at 126 participants (63 per arm) [12].

The study has received institutional approval under Ethical Clearance number LB.02.01/VII.01/47,374/01/2023 issued by the Ethics Committee of the Faculty of Medicine, Universitas Indonesia and under Ethical Clearance number 89/KEPK-RSUPP/05/2023 issued by the Ethics Committee of Health Research of Persahabatan Hospital. The research was conducted in accordance with the Declaration of Helsinki. Written informed consent was obtained from all participants prior to the start of the trial, and consent for publication was also obtained from all participants involved in the study. This study is registered on 9th October 2024 at clinicaltrials.gov under registration number (NCT06017479|| https://clinicaltrials.gov/study/NCT06017479), research permission issued by Head of the Innovation and Intellectual Property Management Unit at Cipto Mangunkusumo National Referral Hospital under registration number LB.02.01/2.6.1/0151/2023 and by Development and Screening of Health Technology and Research Committee at Persahabatan General Hospital under registration number DL 01.01/DXX.2/3133/2024.

### Study design

This investigation was conducted in a referral hospital in Jakarta, Indonesia, and was a prospective single-center randomized clinical trial with balanced randomization [1:1]. Based on the proportion calculation for two separate groups from prior literature, the sample size for this study was determined using the guidelines provided in “Fundamentals of Clinical Research Methodology”. We determined the overall sample size to be 126 patients. Data were collected using a successive sampling technique until the necessary sample size was attained. Following an eligibility screening, these patients were randomly assigned to one of two groups. The random allocation sequence was generated using a computer program to ensure randomization in a ratio 1:1 to either the fosfomycin group and levofloxacin group. The random allocation sequence was generated by an independent statistician who was not involved in the trial’s conduct or analysis. Participant enrollment was carried out by the clinical research team, who verified eligibility criteria but were blinded to the allocation sequence. An independent team, separate from the corresponding author and contributing authors, implemented the allocation sequence and assigned participants to their respective interventions. This independent team ensured allocation concealment throughout the process to prevent any potential bias. Participants were blinded after assignment to interventions. To maintain blinding, the interventions (500 mg levofloxacin and 3 g fosfomycin) were provided in identical packaging, and the team administering the interventions had no knowledge of the allocation sequence.

Individuals who fulfilled all participation requirements and were not excluded were split into two groups at random: those receiving a single dose of 3 g fosfomycin and those receiving a single dose of 500 mg levofloxacin one hour prior to UDS. Participants who met the eligibility requirements were examined using normal UDS protocol. Three days after UDS, we performed a urinalysis on the patients and monitored them for clinical signs of a UTI.

### Outcome assessment

A urine test that shows leukocyturia (more than five white blood cells per field of vision), a positive result for bacteria, nitrite, and/or leukocyte esterase, or any combination of these, is used to diagnose a urinary tract infection. Urine cultures were taken from individuals who had been diagnosed with UTIs after the results of the urinalysis were obtained and the clinical symptoms had been evaluated.

The study employed descriptive statistics to assess various subject characteristics, including age, gender, and indication for urodynamic testing. To evaluate the association between treatment groups and the occurrence of UTI, chi-square analysis was used. Data were processed using SPSS version 24, and *p* < 0.05 was used to determine statistical significance.

## Results

One hundred and eighty-four patients were assessed for eligibility. Fifty-eight were excluded for the following reasons: proven UTI (*n* = 18), being under 18 years old (*n* = 14), use of a urinary catheter (*n* = 10), prior consumption of antibiotics (*n* = 7), and declining to participate (*n* = 8). The remaining one hundred and twenty-six patients, who were willing to participate and met no exclusion criteria, were randomly divided into two groups. A single dose of 500 mg levofloxacin was administered to 63 patients, and another 63 patients received a single dose of 3 g fosfomycin, both given 1 h before undergoing UDS. Recruitment for the study began on December 30, 2022, and follow-up was completed by March 25, 2024, which also marks the study’s primary and overall completion dates.

**Fig. 1:**
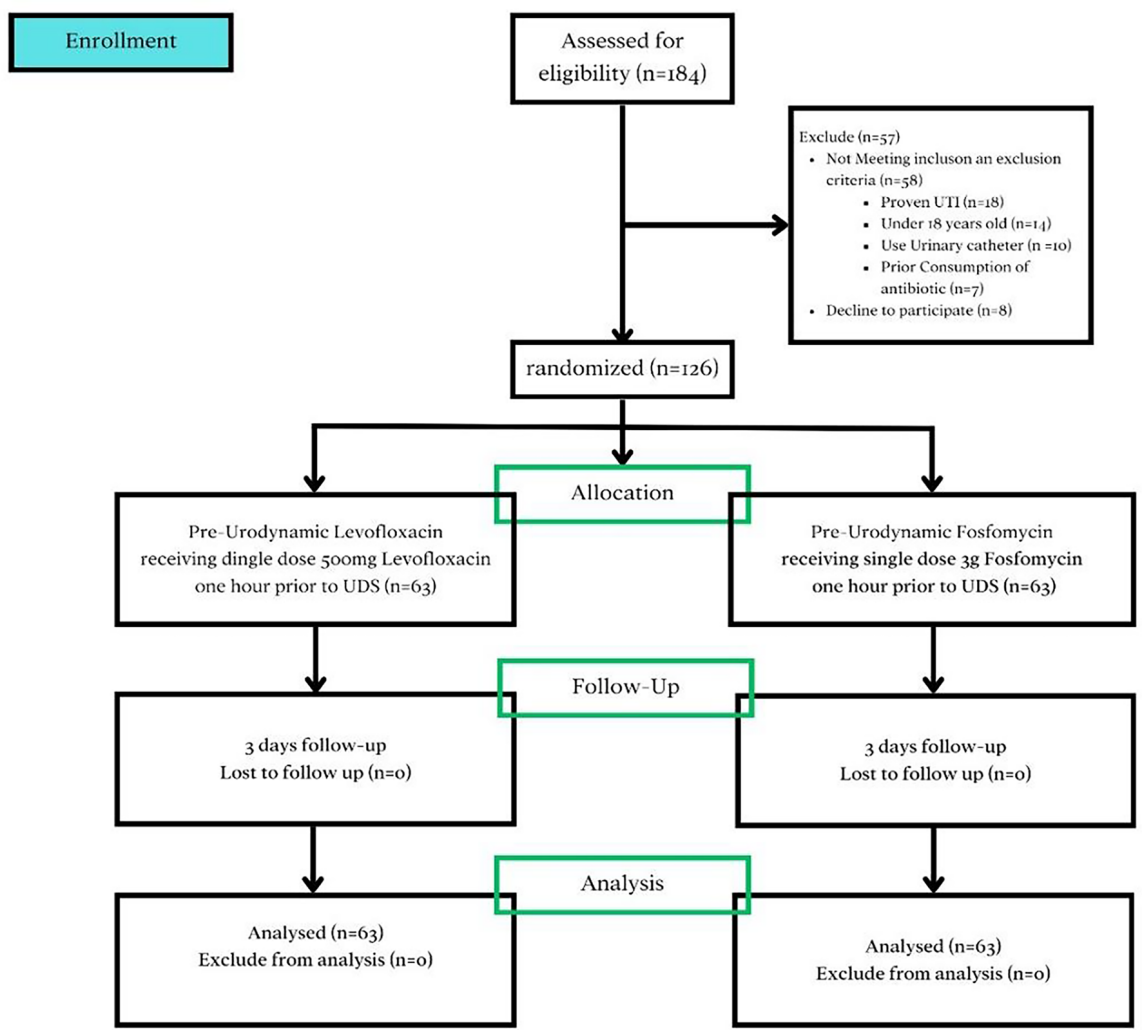
Diagram of the randomized trial on the incidence of urinary tract infections following pre-urodynamic single-dose administration of levofloxacin and fosfomycin

No patient from either group was lost to follow-up, resulting in a total of 126 patients included in the final analysis. A flow diagram of the randomized trial is depicted in Fig. 1. Characteristics of patients who underwent UDS in each group are presented in detail in Table 1. The average age of all participants was 52.2 years. Patients in the levofloxacin group had a mean age of 42 years (SD 17.17), while those in the fosfomycin group had a mean age of 53.99 years (SD 19.08). Based on an independent t-test, there was no statistically significant difference in age distribution between the two groups (*p* = 0.287).

In terms of gender distribution, 75 (58.5%) participants were male and 51 (41.5%) were female. The fosfomycin group comprised 36 males (57.14%) and 27 females (42.86%), whereas the levofloxacin group included 24 males (38.1%) and 39 females (61.9%). Chi-square analysis revealed no significant difference in gender distribution between the two treatment groups (*p* = 0.586).

Regarding clinical indications for UDS, 69 patients (54.8%) had lower urinary tract symptoms (LUTS), 41 (32.8%) had overactive bladder (OAB), 11 (8.9%) had underactive bladder, and 5 (3.5%) had stress urinary incontinence (SUI). Distribution of these diagnoses between the treatment groups is detailed in Table 1. Based on chi-square analysis, there was no statistically significant difference in the distribution of clinical diagnoses between the fosfomycin and levofloxacin groups (*p* = 0.068). This indicates that randomization was generally adequate in balancing baseline clinical characteristics across both groups.

**Table 1:** Subject characteristics

	Receiving Single dose 3 g fosfomycin 1 h prior to UDS (*n* = 63)	Receiving Single dose 500 mg levofloxacin 1 h prior to UDS (*n* = 63)	*P*-Value
Age (years, Mean (SD))	53.99 (19.08)	42 (17.17)	0.287
Gender			
•Male	36 (57.14%)	24 (38.1%)	0.586
•Female	27 (42.86%)	39 (61.9%)	
Clinical diagnosing for performing UDS, n (%)			
•LUTS	41 (32.54%)	28 (22.22%)	0.068
•OAB	18 (14.48%)	23 (18.38%)	
•Underactive Bladder	3 (2.38%)	8 (6.55%)	
•Stress Urinary Incontinence	1 (0.08%)	4 (3.37%)	

* UDS = urodynamic study; UTI = urinary tract infections

In a cohort of 126 patients who underwent UDS, UTI were diagnosed in 26 cases (20.9%)—14 (22.2%) from the levofloxacin group and 12 (19%) from the fosfomycin group—indicating no significant difference in incidence rates between the two groups (*p* = 0.660). The absolute risk reduction (ARR) between the levofloxacin and fosfomycin groups was 3.2% (22.2% − 19%). The relative effect size, expressed as an odds ratio (OR), was 0.8 (95% CI: 0.4–1.95). Of those with UTI, 17 were male—10 from the levofloxacin group and 7 from the fosfomycin group. Symptomatic UTIs were observed in six (4.8%) patients in the levofloxacin group and in seven (5.6%) patients in the fosfomycin group. The relative risk for symptomatic UTI was 1.17 (95% CI: 0.4–3.5), with an ARR of 0.8% (5.6% − 4.8%). No subgroup or adjusted analyses were performed as this was not pre-specified in the protocol. Harms and unintended effects were minimal in both groups. In the fosfomycin group, Three participants (4.76%) in the levofloxacin group reported mild nausea, which resolved without intervention. In the levofloxacin group, two participants (3.17%) reported mild nausea. No serious adverse events were observed during the study period. *E. coli* was the most frequently-identified bacterium in urine cultures, followed by *K. pneumoniae* and *P. aeruginosa*. Additionally, 10 patients with UTI showed no bacterial growth in their urine culture/isolation. The comparison of UTI cases between both groups is described in Table 2.

**Table 2:** Patients’ characteristics

	Receiving Single dose 3 g fosfomycin 1 h prior to UDS (*n* = 63)	Receiving Single dose 500 mg levofloxacin 1 h prior to UDS (*n* = 63)	OR (95% CI)	*P* Value
Number of UTI Cases post-UDS, n (%)	12 (9.52%)	14 (11.38%)	0.8 (0.4–1.95)	0.660
Symptomatic UTI n (%)	7 (5.56)	6 (4.76%)	1.7 (0.5–5.4)	0.380
Positive urine culture n (%)	5 (3.96%)	8 (6.62%)	0.5 (0.1–1.6)	0.225
Type of bacteria found in urine culture, n				
•*E. coli*	2	4		
•*K. pneumoniae*	1	2		
•*P. aeruginosa*	1	2		
•No Growth	8	6		

* UDS = Urodynamic Study; UTI = Urinary Tract Infections OR = Odd Ratio

## Discussion

The purpose of a UDS examination is to find anomalies in the lower urinary tract. A urinary catheter must be inserted for this invasive treatment. Despite the use of aseptic procedures prior to the examination, the risk of developing a UTI afterwards remain [13]. E. coli is the most common etiologic pathogen causing post-UDS UTI [14, 15]. Given this risk, the use of prophylactic antibiotics is recommended to reduce the incidence of UTI following UDS. The American Urology Association (AUA) advises the administration of a single-dose trimethoprim-sulfamethoxazole (TMP-SMX) prior to the urodynamic testing procedure, with first- or second-generation cephalosporin as an alternative antimicrobial prophylaxis [16]. Similarly, the European Association of Urology (EAU) guidelines suggest that the choice of antibiotic should be guided by local resistance patterns and patient-specific factors. Common options include fluoroquinolones or cephalosporins, with adjustments based on local bacterial resistance data [17]. In previous studies conducted in several hospitals in Indonesia, a single dose of 500 mg levofloxacin has been the preferred choice before performing UDS [5]. A study by the Clinical Pathology Department of Dr. Cipto Mangunkusumo National Referral Hospital showed that fosfomycin has better sensitivity compared to levofloxacin against *E. coli* isolates [8]. This is particularly relevant because levofloxacin has been the primary antibiotic used as prophylaxis prior to urodynamic studies in Indonesia, as supported by previous clinical trials [5]. However, due to the higher sensitivity profile of fosfomycin among Indonesian patients, as shown in the local microbiological surveillance data, it was selected as a comparator in this study. Trimethoprim-sulfamethoxazole (TMP-SMX) and nitrofurantoin, although commonly used in international guidelines, were not included in this study because they are not routinely used as prophylactic antibiotics for UDS in Indonesia and have shown lower local sensitivity profiles. Therefore, this study was designed to directly compare fosfomycin and levofloxacin as single-dose prophylactic regimens to assess their effectiveness in reducing the incidence of post-UDS urinary tract infections.

Updated guidance from the European Association of Urology (2024) does not recommend routine antimicrobial prophylaxis for urodynamic testing [16]. In contrast, the American Urological Association (2020 Best-Practice Statement) recommends prophylaxis for selected high-risk patients, including those with neurogenic lower urinary tract dysfunction, immunosuppressed individuals (such as transplant recipients), patients with known or suspected abnormalities of the urinary tract who have undergone recent genitourinary instrumentation, and those recently exposed to antimicrobials, as these groups are considered at increased risk for urinary tract infection (UTI) [17]. These recommendations were developed based on evolving evidence and not in isolation from prior studies.

A 2021 meta-analysis by Wu X-Y et al., which included 1,789 participants across twelve randomized trials, demonstrated that prophylactic antibiotics significantly reduced post-UDS bacteriuria (RR 0.42, 95% CI 0.30–0.60) and decreased the incidence of symptomatic urinary tract infections by approximately one-third (RR 0.65, 95% CI 0.48–0.88). The findings were consistent across studies, showing minimal heterogeneity and supporting the short-term benefit of antimicrobial prophylaxis in select patient populations [29]. 

These results were further supported by a female-focused systematic review conducted by Benseler et al. in 2020. The review analyzed three randomized controlled trials involving a total of 325 women and found that antibiotic prophylaxis may reduce the rate of bacteriuria after urodynamic studies (pooled 5/206 vs. 8/119; OR ≈ 0.47). However, due to very low event counts, the effect on symptomatic urinary tract infections remained inconclusive (2/206 vs. 3/119). A sub-analysis of the individual studies identified levofloxacin, trimethoprim-sulfamethoxazole, and nitrofurantoin as the most effective agents, with levofloxacin in particular reducing bacteriuria cases from 6/63 in the placebo group to 1/59 in the prophylaxis group [30]. 

Local evidence echoes these findings: a randomized controlled trial by Rahardjo et al. (2016) conducted in our own setting demonstrated a markedly lower post-UDS UTI rate with levofloxacin prophylaxis (12.7%) versus placebo (28.6%; *p* = 0.028), against an overall baseline incidence of 20.6%.^(5)^ Although the newest EAU guidance advises against fluoroquinolone prophylaxis because of growing resistance, levofloxacin was retained in our protocol because, at the time of study initiation, it was the agent most widely available, supported by local susceptibility patterns, and familiar to practicing clinicians [8]. 

Taken together, the global evidence from Wu et al., the sex-specific analysis by Benseler et al., and our own institutional RCT provided a cogent rationale for incorporating single-dose levofloxacin prophylaxis when this study was launched. Our results now offer context-specific data that can guide Indonesia’s gradual alignment with updated international recommendations while addressing the practical realities of local healthcare delivery.

A study by Raharjo et al. previously found an incidence rate of UTI at 19.8%, which did not differ significantly from the incidence rate observed in the current study, at 20.9%.^(5)^ The incidence rate of post-urodynamic UTI in previous studies varies widely, ranging from 1.5–36%.^(2–3)^ This may be attributed to differences in the duration of the examination, catheter insertion techniques, differences in the study population, including age, risk factors for post-UDS UTI, differences in UDS examination techniques and methods, and differences in the operational definitions and criteria used to establish the diagnosis of UTI [18–20]. 

Several studies did not support the routine use of antibiotic prophylaxis, with findings showing no significant difference in the occurrence of post-UDS UTI between patients who received prophylactic antibiotics and those who did not [21, 22]. On the other hand, 500 mg levofloxacin administered over three days decreased the occurrence rate compared to placebo from 28.6 to 12.7%, according to the study by Rahardjo, et al. [4]. This evidence highlights the clinical benefit of administering antibiotics prior to UDS in reducing the risk of UTI in our center. Therefore, in the present study, a placebo arm was not included, as it would have been ethically inappropriate to withhold prophylactic antibiotics given prior findings of significant benefit over placebo. Instead, this randomized controlled trial was designed to compare two active regimens—fosfomycin and levofloxacin—to determine which agent is more effective and potentially safer as a single-dose prophylactic option.

Other studies observed no discernible difference between taking 500 mg of levofloxacin once a day for three days versus giving it as a single dosage. However, a single dose is recommended due to being more cost-effective, simpler, and reducing the potential for antibiotic resistance [5]. The administration of prophylactic antibiotic for UDS is recommended, as it is proven to reduce bacteriuria and decrease the risk of UTI [23, 24]. To date, *E. coli* remains the most common cause of UTI, while data from the Clinical Pathology Department of Dr. Cipto Mangunkusumo National Referral Hospital indicate that fosfomycin has better sensitivity compared to levofloxacin against *E. coli* isolates [8]. This is the first randomized trial comparing the prophylactic use of the antibiotics fosfomycin and levofloxacin to lower the incidence of UTIs following UDS.

Administering a single dose of 500 mg of levofloxacin one hour prior to the UDS examination and a single dose of 3 g fosfomycin as prophylactic antibiotic do not significantly alter the clinical outcomes. Although the odds ratio (OR 0.8 ± 0.4–1.95) suggested a lower likelihood of UTI with fosfomycin administration (*n* = 12; 9.52%) compared to levofloxacin administration (*n* = 14; 11.38%), this difference was not statistically significant. The study also indicated that the fosfomycin group (*n* = 7; 5.56%) experienced more symptomatic post-UDS UTI compared to the levofloxacin group (*n* = 6; 4.76%). However, when urine cultures were performed, the fosfomycin group had fewer positive bacterial cultures compared to the levofloxacin group, albeit the difference being not statistically significant (*p* = 0.660). These findings contradict data reported by the Clinical Pathology Department of Dr. Cipto Mangunkusumo National Referral Hospital, which indicated that fosfomycin has better sensitivity against *E. coli* isolates compared to levofloxacin [8]. A study conducted by Fajfr et al. also showed that oral fosfomycin provided better outcomes than levofloxacin [25]. Other studies have also suggested that fosfomycin is a non-inferior option to other antibiotics in patients with UTI [26, 27]. The results from this study are consistent with the findings of Wang et al., who observed no difference in clinical outcomes between single-dose fosfomycin and other types of antibiotics, including fluoroquinolones [28]. 

This study was conducted in two referral hospitals, Cipto Mangunkusumo National Referral Hospital and Persahabatan General Hospital, which improves its generalizability compared to single-center studies. However, the findings may still not be fully generalizable to other healthcare settings or regions with differing bacterial resistance patterns or resource availability. Additionally, the sample size may have led to imprecision in effect estimates, particularly for subgroup outcomes. No adjustments were made for multiple comparisons, which should be considered when interpreting the findings.

Although no statistically significant difference was observed, the trend toward fewer positive bacterial cultures in the fosfomycin group suggests a possible clinical benefit that may warrant further study. These findings should be interpreted in the context of balancing benefits, such as bacterial sensitivity, and harms, including potential antibiotic side effects and resistance. The current national health insurance system in Indonesia does not yet cover fosfomycin as an antibiotic of choice, especially according to indications for the treatment of non-complicated acute lower urinary tract infections and prevention of urinary tract infections in trans-urethral diagnostic procedures. Therefore, in line with the results of this randomized trial, levofloxacin remains a suitable treatment option for the Indonesian population. Until this article was written, there had been no cost-effectiveness analysis conducted to compare levofloxacin and fosfomycin.

Although the study offers useful findings, its limitations should be taken into account when interpreting the results. First, the trial was restricted to non–high-risk patients, as defined by the exclusion criteria. Therefore, different results may be observed in populations with a broader or higher-risk profile. Second, the study was conducted at two tertiary referral centers in Jakarta, which may limit the generalizability of the findings to other healthcare settings with differing antimicrobial resistance patterns or resource availability. Third, the follow-up period was limited to three days post-procedure, potentially missing late-onset infections or complications. Finally, the study did not assess cost-effectiveness or the long-term impact of antibiotic use on antimicrobial resistance, both of which are important considerations in guiding clinical practice.

## Conclusion

There was no significant difference in clinical outcomes following the administration of a single dose 3 g fosfomycin and a single dose 500 mg levofloxacin as prophylactic antibiotics one hour before UDS examination in our center. Further research is required through multicenter and multinational studies, as bacterial profiles and antibiotic resistance patterns may vary between regions and healthcare settings.

## Electronic supplementary material

Below is the link to the electronic supplementary material.

Supplementary Material 1

## Data Availability

The data supporting the findings of this study, “Comparison of the Effectiveness of Single-Dose Levofloxacin with Single-Dose Fosfomycin Pre-Urodynamic Study Related to the Incidence of Urinary Tract Infection: A Randomized Controlled Trial,” are provided within the manuscript and its supplementary information files. Further requests for data can be directed to the corresponding author, Andika Afriansyah, M.D., Division of Urology, Department of Surgery, Persahabatan Hospital, Faculty of Medicine, Universitas Indonesia.

## Declarations

### Study registration date

2023-08-24.

The study was registered with ClinicalTrials.gov under registration number NCT06017479. The full trial protocol is available on ClinicalTrials.gov (NCT06017479) or as Supplementary Material attached to this article. This study adheres to the CONSORT guidelines for reporting randomized controlled trials. A completed CONSORT checklist has been provided as a supplementary file.
